# Left ventricular phosphorylation patterns of Akt and ERK1/2 after triiodothyronine intracoronary perfusion in isolated hearts and short-term *in vivo* treatment in Wistar rats

**DOI:** 10.22038/ijbms.2020.44776.10451

**Published:** 2020-08

**Authors:** José A. Morales, Ruth M. López, Jorge S. López, Jair Lozano, Rosa A. Jarillo, Héctor Flores, Enrique F. Castillo

**Affiliations:** 1 Sección de Estudios de Posgrado e Investigación, Escuela Superior de Medicina, Instituto Politécnico Nacional, Ciudad de México, México; 2 Departamento de Biología Celular, Instituto Nacional de Perinatología, Ciudad de México, México; 3 Departamento de Inmuno-Bioquímica, Instituto Nacional de Perinatología, Ciudad de México, México

**Keywords:** Akt, ERK1-2, Heart hypertrophy, Rat, Triiodothyronine treatment

## Abstract

**Objective(s):**

To determine the effects of triiodothyronine (T3) intracoronary perfusion in isolated hearts and short-term administration in rats on the left ventricular (LV) phosphorylation patterns of Akt and ERK1/2.

**Materials and Methods:**

Cardiodynamic and hemodynamic parameters were evaluated in Langendorff–perfused hearts. Left ventricles were used for histomorphometric and Western blot analyses. Short-term hyperthyroidism was established by T3 (500 μg.kg^-1^.d^-1^; subcutaneous injection) for 1 (T3_1d_), 3 (T3_3d_), and 10 (T3_10d_) days.

**Results:**

Isolated hearts receiving T3 perfusion did not modify LV developed pressure, +dP/dt_max_, -dP/dt_min_, heart rate, and coronary perfusion pressure compared with vehicle-perfused hearts. P-ERK1/2 and p-Akt levels in LV tissues after 5, 15, or 60 min of T3 or vehicle perfusion were similar. Compared with their time-matched controls, isolated hearts of T3_3d_ and T3_10d_ rats exhibited LV hypertrophy and increased absolute values of +dP/dt_max_ and -dP/dt_min_ (i.e., positive inotropic and lusitropic effects). P-ERK1/2 decreased in LV tissues of T3_1d_ and T3_10d_ but not in those of T3_3d_ rats, and p-Akt levels augmented in left ventricles of T3_3d_ and stayed unaltered in those of T3_1d_ and T3_10d_ rats.

**Conclusion:**

T3 intracoronary perfusion did not alter cardiodynamics and hemodynamics nor influence the activation of Akt and ERK of normal hearts. Accordingly, the rapid non-genomic effects of T3 were not evident. Short-term T3 treatment provoked cardiac hypertrophy coincidental with increased LV function and associated with transient Akt activation and cyclic ERK1/2 inhibition; which implies activation of physiological hypertrophy signaling and deactivation of pathological hypertrophy signaling, respectively.

## Introduction

Thyroid hormones (THs), 3, 5, 3’, 5’ tetraiodo-L-thyronine (T4) and 3, 5, 3’ triiodo-L-thyronine (T3), exert cardinal effects on the cardiovascular system. Abnormal increases in THs levels – observed in hyperthyroidism – promote an overactive cardiovascular state with augmented cardiac output and reduced peripheral vascular resistance (1, 2). Interestingly, the increased activity of THs generates cardiac hypertrophy and angiogenesis, which keeps pace with the enlarging heart (3, 4).

**Figure 1:**
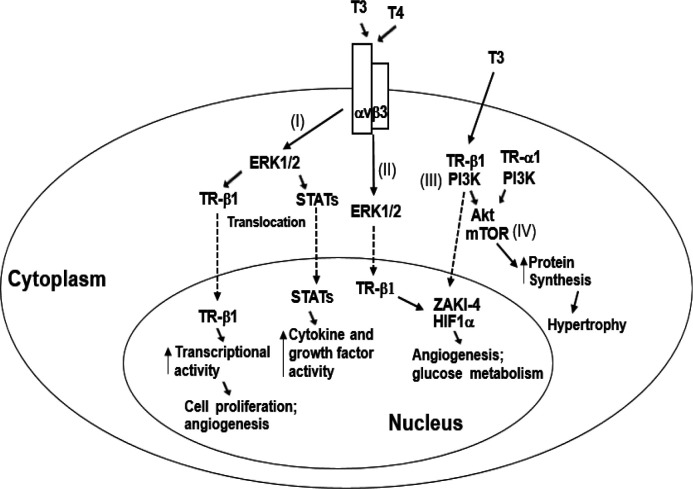
Schema showing non-genomic actions of thyroid hormones started at the plasma membrane receptors on integrin αvβ3, or 3, 5, 3’ triiodo-L-thyronine receptors localized in the cytoplasm, entailed in their cardiovascular effects. (I) Through integrin αvβ3, T4, and T3 stimulate the ERK1/2 signaling pathway, producing TR-β1 and STAT-1α phosphorylation and translocation to the nucleus where its transcriptional activity is heightened (5, 6). (II) Thyroid hormone-stimulated ERK1/2 can also move to the nucleus to phosphorylate nuclear TRβ1. Complex cellular events induced by the cell surface receptor include angiogenesis and cell proliferation (5, 6). (III) T3 may also activate cytoplasmic PI3K and initiate downstream transcription of specific genes, such as ZAKI-4, a calcineurin inhibitor, and hypoxia-inducible factor-1α (HIF1α) (5, 6). (IV) In the cytoplasm, T3 binding to TRα1 and TRβ1 activates the PI3K/Akt/mTOR signal transduction pathway, increasing protein synthesis and causing activation of a hypertrophic gene program (3).

T4: 3, 5, 3', 5' tetraiodo-L-thyronine; T3: 3, 5, 3’ triiodo-L-thyronine; PI3K: phosphatidylinositol 3-kinase; Akt: protein kinase B; MAPK: mitogen-activated protein kinase; ERK1/2: extracellular signal-regulated kinases; ZAKI-4: an anti-calcineurin; HIF1α: hypoxia-inducible factor-1α; TR: thyroid hormone receptor; STAT: signal transducer and activator of transcription; mTOR: mammalian target of rapamycin.

THs induce their effects through genomic mechanisms by binding to receptors inside the nucleus and regulating gene transcription, or through non-genomic mechanisms distinguished as those initiated outside the cell nucleus (5-7). The non-genomic actions of THs require plasma membrane receptors and receptors located in the cytoplasm; notwithstanding, THs actions initiated in the cytoplasm or plasma membrane have the potential to influence gene expression (5, 6). On this matter, the signaling transduction pathways that are involved in the cardiovascular effects of THs have not been satisfactorily elucidated. Nevertheless, among others, pro-survival signaling pathways such as phosphatidylinositol 3-kinase (PI3K)/protein kinase B (Akt) and mitogen-activated protein kinase (MAPK)/extracellular signal-regulated kinases 1/2 (ERK1/2) have been implicated in the cardiovascular effects of THs; importantly, in the development of cardiac hypertrophy and angiogenesis, and upholding cellular survival (3-5, 8-10) (for a schema of these pathways, see Figure 1). 

The evidence supporting the activation by THs of the signaling pathways mediated by Akt and ERK1/2 in the heart comes predominantly from studies with cardiac tissues obtained from animals treated short-term or long-term with these hormones (1-11). In hyperthyroid animals, most studies were defined by a unique period of treatment; that is, the temporal evolution of changes in expression or activation of the molecules in question was not characterized. In this regard, however, we found one important temporal course study, developed with hearts of T4 treated mice for 3, 7, 14, or 30 days (12). In the left ventricles of hyperthyroid mice, the results suggested a transient activation of Akt (between 3 and 14 days) as a causal factor of cardiac hypertrophy and described ERK1/2 late activation (at 30 days) not associated with a specific effect (12). Thus, short-term transient activation of Akt was established without alterations in ERK1/2 activity, followed by belated activation of ERK1/2 without coincident modification in Akt function (12). Nevertheless, when studies in the hearts of mice treated at single intervals of time with THs are considered, some results showed that short-term (14 days) THs treatments may inhibit ERK activation in normal cardiac tissues and injured myocardium (13). In T4 treated mice for 2 wk, ventricular cardiac tissues presented decreased p-ERK levels with respect to control tissues (13). Moreover, 2 wk T3 treatment reduced p-ERK1/2 in pressure-overloaded hypertrophied mice hearts, somehow by activating the PI3K/Akt pathway (13); thereby, T3-induced short-term inhibition of ERK1/2 linked to the activation of Akt was recognized (13). These controversial works (12, 13) exemplify that the temporal evolution of Akt and ERK1/2 activations has not been satisfactorily clarified in hearts from experimental hyperthyroid animals. On the other hand, in one acute temporal course study in rat isolated hearts subjected to ischemia/reperfusion, T3 administration at the time of reperfusion improved postischemic recovery of function while limiting apoptosis in the time frame of 60 min; however, the levels of ERK1/2 phosphorylation were not modified and those of Akt decreased transiently but its significance was not explained (14). Noticeably, we could not find studies on isolated hearts of normal animals where the intracoronary perfusion of T3 had been characterized in its capacity to modify the phosphorylation levels of Akt and ERK1/2.

Based on the foregoing analysis, it is clear that the temporal evolution of Akt and ERK1/2 activation has not been adequately explained in hearts after acute *in vitro* and short-term *in vivo* thyroid hormone treatments. We contributed to elucidating this problem: characterizing the rat heart phosphorylation patterns of Akt and ERK that could be induced by T3, in the order of minutes and days, related them appropriately to myocardial and coronary functions and hypertrophy.

The main aim of this temporal course study was to investigate the effects of acute T3 intracoronary perfusion in isolated rat hearts and short-term *in vivo* T3 administration in Wistar rats, on the left ventricular phosphorylation patterns of PKB/Akt and ERK1/2. Complementary cardiodynamic and hemodynamic data were obtained with isolated hearts, receiving acute intracoronary perfusion of T3 or after short-term T3 *in vivo* treatment. Cardiac hypertrophy development was studied in the left ventricles of short-term T3 treated rats.

## Materials and Methods

All experimental procedures were approved by the Animal Care and Use Committee of our institution and complied with the Guide for the Care and Use of Laboratory Animals (1996, National Academy Press, Washington, DC, USA) and the National Health and Medical Research Council of México guidelines.

***General procedures***

Male Wistar rats (250–300 g body mass, 10–12 weeks old) were housed under controlled conditions (22±2 ^°^C, 60±5% humidity and artificial light from 06:00 to 18:00 hrs); normal chow and tap water were given *ad libitum*. Body mass was recorded daily. The rats were randomly separated into different treatment groups.

***Short-term T3-induced experimental hyperthyroidism***

Based on previous research (12, 15), short-term experimental hyperthyroidism was induced by subcutaneous daily injection of T3 (Sigma Chemical Company; MO, USA) (500 **μ**g.kg^-1 ^diluted in alkaline saline solution: 0.5 mM NaOH in 0.9% NaCl) for 1 (T3_1d_), 3 (T3_3d_), and 10 (T3_10d_) days. The control animals were injected with the vehicle (V) at the same volume (1 mL.kg^-1^) for 1 (V_1d_), 3 (V_3d_), and 10 days (V_10d_). The experiments were performed 24 hr after the last dose of T3 or V. 

***Assessment of the hyperthyroid state***

The hyperthyroid state was determined in all short-term T3 treated groups, as described formerly (16). Blood samples were collected without anticoagulant from abdominal aortas, centrifuged at 1000 *g* for 10 min at 4 ^°^C, and stored at - 20 ^°^C until assay. Levels of thyroid-stimulating hormone (TSH) and free T3 (fT3) were determined in the serum of control and hyperthyroid groups using enzyme-linked immunosorbent assay (ELISA) kits (TSH: ALPCO, Salem, NH, USA; fT3: Monobind, Inc., Lake Forest, CA, USA) according to manufacturer’s specifications. 

***Determination of left ventricular hypertrophy***

Left ventricular dry mass (LVdM) to tibia length (TL) ratio, LVdM/TL (mg/mm), was used as an index of cardiac hypertrophy, regularly observed in hyperthyroid disorder (17).

Six rats from each experimental group were included in the histological analysis. Freshly dissected left ventricular tissues were fixed in 4% paraformaldehyde, dehydrated, and embedded in paraffin. 5 µm thick sections were cut and stained either with Hematoxylin and Eosin to measure cardiomyocytes cross-sectional areas or with collagen specific Sirius Red for examining fibrosis formation (18). The mean cross-sectional areas of 300–360 randomly selected cardiomyocytes of each experimental group were measured using the ImageJ software (National Institutes of Health, Bethesda, MD, USA). To calculate the mean interstitial collagen, 60 randomly chosen frames of each experimental group were assessed using the ImageJ software. The positively stained (red) collagen area was expressed as a percentage of the total area (18). 

***Isolated langendorff heart preparations***

These experiments were developed with isolated hearts after short-term *in vivo* T3 or vehicle treatments; as well, acute intracoronary perfusion of T3 or vehicle in isolated hearts from normal rats was characterized. 

Rats were anesthetized with sodium pentobarbital (50 mg·kg^-1^, IP) and then injected subcutaneously with heparin (100 U·kg^-1^). Fifteen minutes after heparin injection, rats were sacrificed, and hearts were immediately removed and immersed in cold Krebs–Henseleit solution (KHS). Each isolated heart was perfused retrogradely, through an aortic stump at a constant flow rate (10 ml·min^-1^). A constant flow setup allowed us to examine the effect of T3 treatments on coronary vasomotor tone; a parameter which can be derived from coronary pressure using Ohm’s law (19). The perfusion was performed by using a KHS containing (mM): NaCl (118), KCl (4.7), CaCl_2_ (1.5), MgSO_4_ (1.2), NaHCO_3_ (25), KH_2_PO_4_ (1.2), dextrose (11), and bi-distilled water, equilibrated with continuously gassing 95% O_2_ and 5% CO_2_ at 37 °C, to yield a physiological pH of 7.4. After a stabilization period of 30 min, the cardiac functions were determined by a modified isovolumetric Langendorff technique (19-21). The left ventricular pressure was measured with a water-filled balloon constructed of plastic film (3–5 mm in diameter) inserted into the left ventricle via the left atrium and connected to a pressure transducer (TSD104A, Biopac Systems Inc., Santa Barbara, CA, USA) coupled to a software (Acqknowledge program; MP 100WSW, Biopac Systems Inc.) for data acquisition. The volume of the balloon was adjusted to produce an end-diastolic pressure of 8–10 mmHg in all groups. Measurements were carried out by the left ventricular developed pressure (LVDP), the first derivatives of ventricular pressure maximum positive (+dP/dt_max_) and minimum negative (−dP/dt_min_), and heart rate (HR). Coronary perfusion pressure (CPP) was measured through a lateral connection in the perfusion cannula connected to a pressure transducer (TSD104A, Biopac Systems Inc.). When the effects of T3 intracoronary perfusion were investigated in isolated hearts, T3 was perfused at either 1 nM or 100 nM, during 1 hr in different hearts. Vehicle intracoronary perfusion (vehicle concentration needed to dissolve T3 either 1 nM or 100 nM) was used as a control in distinct hearts. T3 and vehicle were injected into the perfusion cannula with an infusion pump at a constant rate. In a constant flow arrangement, the flow of the perfusate through the coronary vasculature is maintained at a constant rate (using a peristaltic pump), allowing to monitor any changes in coronary pressure during the experiment (21).

***Western blot analysis***

The analysis was performed with isolated hearts from all groups of short-term *in vivo* T3 treated rats and their matching temporal controls. For acute intracoronary perfusion experiments, the isolated hearts obtained from normal rats were previously perfused during either 5, 15, or 60 min with T3 (100 nM) or the corresponding concentration of vehicle; subsequently, the hearts were prepared for Western blot assay. 

Western blot was implemented as described formerly (22). Tissue samples were prepared from a collection of 4 hearts per group. Left ventricular tissues were separately immersed in liquid nitrogen and stored at - 80 ^°^C until analysis. The frozen tissues were thawed, minced into small pieces, and homogenized with a polytron homogenizer in Tris-HCl, pH 7.4, with phosphatase and protease inhibitors cocktail (PhosSTOP, cOmplete Mini, Roche, Germany). Lysis buffer was chosen to ensure effective sample solubilization for protein electrophoresis; protease and phosphatase inhibitors were added to lysate samples to protect proteins of interest and to preserve the phosphorylation state of these, respectively. Homogenates were centrifuged (14000 *g* for 20 min at 4 °C) and supernatant protein levels were assayed (Lowry method). The solubilized samples were subjected to SDS-PAGE in 10% polyacrylamide gel (50 µg of protein was loaded per well). After electrophoresis, proteins were electrotransferred onto a polyvinylidene fluoride membrane (Hybond-P PVDF, Amersham Biosciences, NJ, USA) at 15 V for 45 min (Transblot SD, Bio-Rad Laboratories, Inc.; CA, USA). The membrane was soaked in Tris-buffered saline (TBS: 10 mM Tris-HCl, 150 mM NaCl) containing 5% non-fat dry milk and 0.1% polyoxyethylene-sorbitan monolaurate (Tween 20), for 2 hr at room temperature, and then incubated with a primary antibody overnight at 4 ^°^C. Membranes were probed for phospho-Akt (ser473; 1:1000), Akt (1:4000), phospho-ERK1/2 (Thr202/Tyr204; 1:1000), ERK1/2 (1:2000) (Cell Signaling Technology, Danvers, MA, USA), and β-actin as the loading control (1:3000) (Santa Cruz Biotechnology, Santa Cruz, CA, USA). The blotted membranes were washed and incubated with a peroxidase-conjugated goat anti-rabbit secondary antibody (1:10000 dilution) for 1 hr at room temperature (Zymed Laboratories, Inc., San Francisco, CA, USA). Immunoreactivity was visualized with enhanced chemiluminescence (ECL) Western blotting detection luminol reagent (Clarity TM Western ECL Substrate, Bio-Rad Laboratories, USA). Images were digitally acquired from films, and densitometric analysis was performed using the Quantity One Image Acquisition and Analysis Software (Bio-Rad Laboratories, Inc.). Data are reported as normalized absorbance. 

***Statistical analysis***

Results are expressed as means±SEM for the number (n) of samples obtained from 4-8 different rats. Statistical analysis was done by using either one-way, repeated measures, or two-way analysis of variance (ANOVA) followed by Tukey, Dunnett, or Sidak test, as appropriate (Prism version 6.0, Graph Pad Software; San Diego, CA, USA). In all comparisons, a value of *P*<0.05 was statistically significant.

## Results

***3, 5, 3’ triiodo-L-thyronine treatment-induced hyperthyroidism in rats***

**Figure 2:**
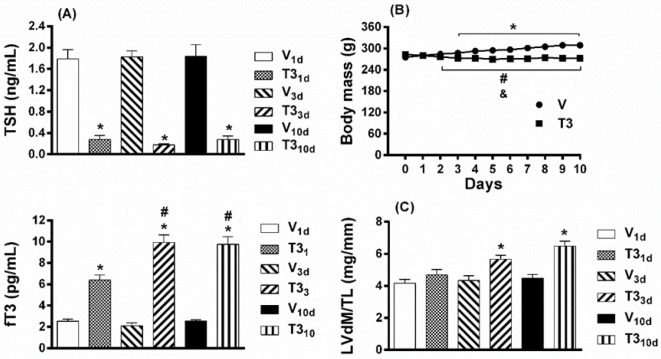
Characteristics of short-term hyperthyroidism. (A) Serum levels of TSH and fT3 in short-term hyperthyroid rats. (B) Body mass values of rats with short-term hyperthyroidism. (C) LVdM/TL ratio as an indicator of cardiac hypertrophy in rats with short-term hyperthyroidism. Short-term hyperthyroidism was induced by a daily subcutaneous injection of T3 (500 μg.kg^-1^) for 1 (T3_1d_), 3 (T3_3d_), and 10 (T3_10d_) days. Control animals were injected daily with the vehicle for 1 (V_1d_), 3 (V_3d_), and 10 (V_10d_) days. Values are means±SEM. (A) *P<0.05 vs V respective in time;^ #^*P*<0.05 vs T3_1d_; n=8 rats per group (one-way ANOVA with Tukey’s *post hoc* test). (B) *P<0.05 T3 vs V; n=38 animals for each group (two-way ANOVA with Sidak’s post hoc test); ^#^*P*<0.05 T3 compared with day 0; ^&^*P*<0.05 V compared with day 0 (repeated measures ANOVA with Dunnett’s *post hoc* test). (C) *P<0.05 vs V respective in time; n=8 hearts per group (one-way ANOVA with Tukey’s *post hoc* test)

T3: 3, 5, 3’ triiodo-L-thyronine; TSH: thyroid-stimulating hormone; fT3: free T3; LVdM/TL: left ventricular dry mass to tibia length ratio; V: vehicle

The serum levels of fT3 increased, and those of TSH decreased, significantly in T3_1d_, T3_3d,_ and T3_10d_ rats in respective relation with V_1d_, V_3d_, and V_10d _animals (Figure 2A). These data can be considered indicative of the hyperthyroid state of the rats treated with T3. Serum concentrations of fT3 did not differ statistically between T3_3d_ and T3_10d_ rats, although both groups showed a significant increase in fT3 in relation to T3_1d_ rats (Figure 2A). Thus, it is possible to appreciate a rise in the intensity of the hyperthyroid state in the third and tenth days of T3 treatment. However, TSH concentrations did not vary in a significant way between the groups of T3 treated rats (Figure 2A). 

The body mass of rats treated short-term *in vivo* with T3 was significantly less than that of control rats from day three to tenth (Figure 2B). Also, T3-treated rats had reduced body mass, and in those treated with vehicle it increased, from the 2^nd^ day in comparison with their respective values at the beginning of treatments (day 0; Figure 2B). The stimulatory effects of THs on metabolic activity can explain the loss of body mass (23). 

***Short-term experimental hyperthyroidism elicited left ventricular hypertrophy***

**Figure 3:**
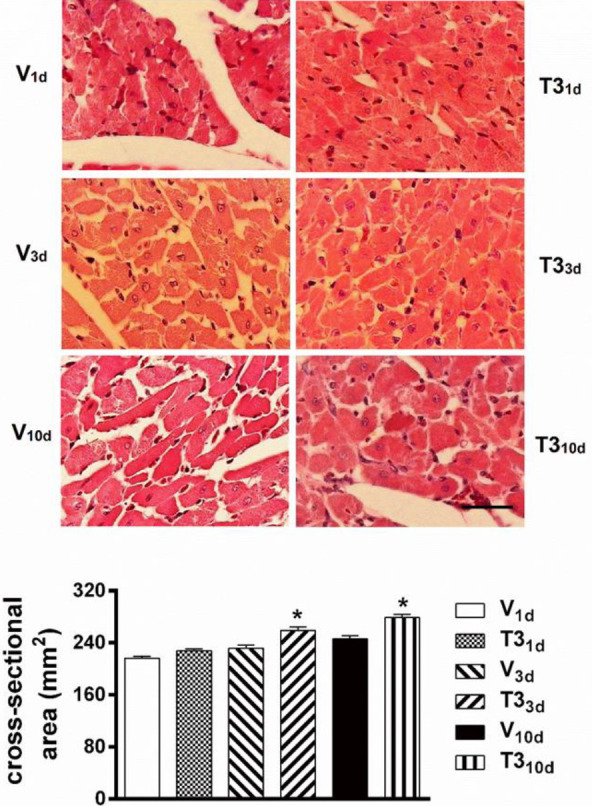
Cardiomyocytes hypertrophy in short-term 3, 5, 3’ triiodo-L-thyronine treated rats. (top) Representative microphotographs of histological slices stained with Hematoxylin and Eosin of hearts from short-term T3 treated and vehicle-treated rats (scale bar 25 µm). (bottom) Quantitative analysis of cardiomyocytes sizes in each of the different animal groups. Short-term hyperthyroidism was induced by the daily subcutaneous injection of T3 (500 μg.kg^-1^) for 1 (T3_1d_), 3 (T3_3d_), and 10 (T3_10d_) days. Control animals were injected daily with the vehicle for 1 (V_1d_), 3 (V_3d_), and 10 (V_10d_) days. Values are means±SEM. *P<0.05 vs V respective in time; n=300–360 cardiomyocytes from 6 hearts per group (one-way ANOVA with Tukey’s post hoc test).

T3: 3, 5, 3’ triiodo-L-thyronine; V: vehicle

**Figure 4:**
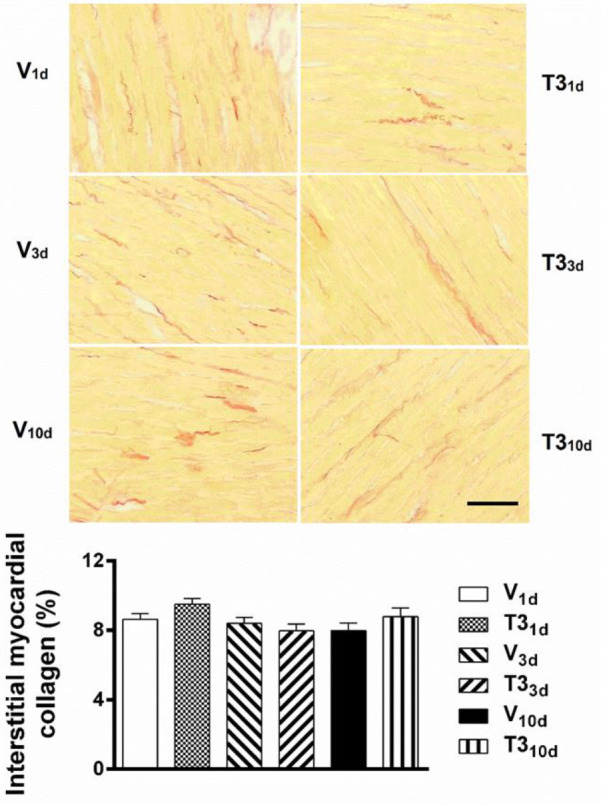
Absence of signs of fibrosis in the left ventricles of 3, 5, 3’ triiodo-L-thyronine treated rats. (top) Representative microphotographs of histological slices stained with Sirius Red of hearts from short-term T3 treated and vehicle-treated rats (red color indicates collagen fibers; scale bar 25 µm). (bottom) Quantitative analysis of left ventricular collagen-positive areas in each of the different animal groups. The positively stained collagen area was expressed as a percentage of the total area. Short-term hyperthyroidism was induced by the daily subcutaneous injection of T3 (500 μg.kg^-1^) for 1 (T3_1d_), 3 (T3_3d_), and 10 (T3_10d_) days. Control animals were injected daily with the vehicle for 1 (V_1d_), 3 (V_3d_), and 10 (V_10d_) days. Values are means±SEM. *P* not significant; n= 60 randomly chosen frames from 6 hearts per group (one-way ANOVA)

T3: 3, 5, 3’ triiodo-L-thyronine; V: vehicle

To define the development of T3-induced cardiac hypertrophy, experimental hyperthyroidism of 1, 3, and 10 days of duration was established in rats. T3_1d_ rats showed no significant increase in LVdM/TL ratio, but T3_3d_ and T3_10d_ rats exhibited increases in LVdM/TL, both significant, compared with their respective time-based controls (Figure 2C). Consistent with these results, the cardiomyocytes cross-sectional areas were greater in T3_3d_ and T3_10d_ hearts, in comparison with cardiomyocytes cross-sectional areas of V_3d_ and V_10d_ hearts, respectively (Figure 3). T3_1d_ cardiomyocytes did not show greater cross-sectional areas than V_1d_ cardiomyocytes (Figure 3). Sirius Red staining to determine collagen deposition showed no more connective tissue in the left ventricles of T3_1d_, T3_3d_, and T3_10d_ rats compared with their respective temporal controls (Figure 4). 

***Short-term in vivo T3 treatment enhanced ex vivo cardiac function***

**Figure 5:**
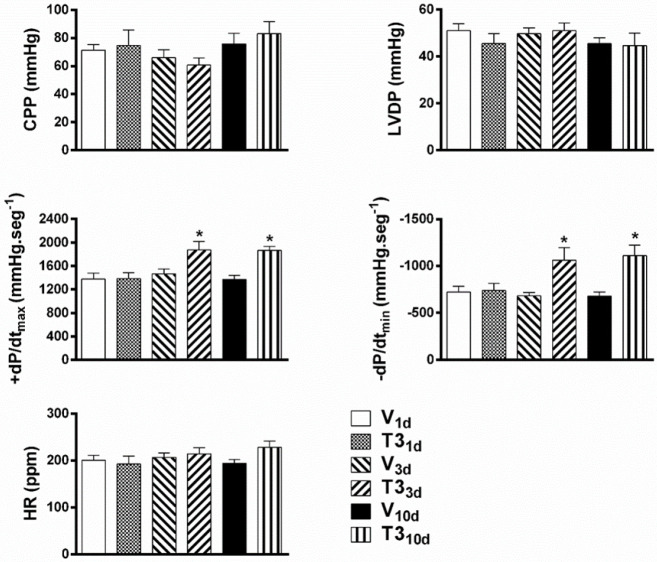
Increased cardiac function in isolated hearts from short-term 3, 5, 3’ triiodo-L-thyronine treated rats. Comparison of the values of LVDP, +dP/dt_max_, -dP/dt_min_, HR, and CPP of isolated hearts from short-term T3 treated and vehicle-treated rats. Short-term hyperthyroidism was induced by the daily subcutaneous injection of T3 (500 μg.kg^-1^) for 1 (T3_1d_), 3 (T3_3d_), and 10 (T3_10d_) days. Control animals were injected daily with the vehicle for 1 (V_1d_), 3 (V_3d_), and 10 (V_10d_) days. Values are mean±SEM. *P<0.05 vs V respective in time; n= 6 hearts per group (one-way ANOVA with Tukey’s *post hoc* test)

T3: 3, 5, 3’ triiodo-L-thyronine; V: vehicle; LVDP: left ventricular developed pressure; HR: heart rate; CPP: coronary perfusion pressure; +dP/dt_max_: first derivative of ventricular pressure maximum positive; (−dP/dt_min_) first derivative of ventricular pressure minimum negative

Isolated hearts of T3_1d_ and V_1d_ rats showed comparable LVDP, +dP/dt_máx_, -dP/dt_min_, HR, and CPP values (Figure 5). The isolated hearts from T3_3d_ and T3_10d_ rats did not show modifications of HR, CPP, and LVDP but exhibited significant augmentations in the maximum rate of pressure increase (+dP/dt_max_) and maximum rate of pressure decrease (-dP/dt_min_) when compared with hearts of V_3d_ and V_10d_ rats, respectively (Figure 5). 

***Lack of effects of 3, 5, 3’ triiodo-L-thyronine intracoronary perfusion to isolated hearts***

**Figure 6:**
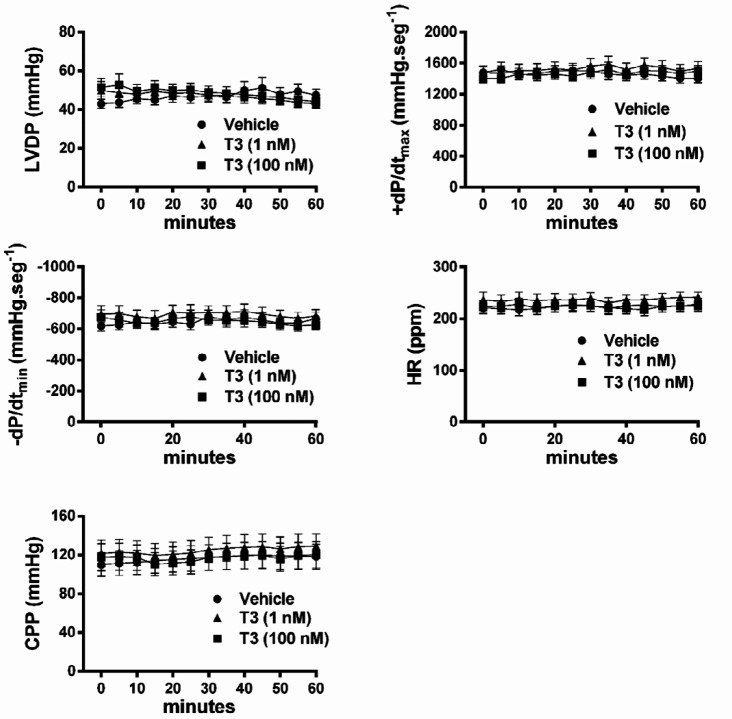
Absence of cardiodynamic and hemodynamic effects of 3, 5, 3’ triiodo-L-thyronine intracoronary perfusion to isolated hearts. Comparison of the values of LVDP, +dP/dt_max_, −dP/dt_min_, HR, and CPP of isolated hearts infused with T3 or vehicle. T3 was perfused at either 1 nM or 100 nM, for 1 hr in different hearts. Control hearts were perfused with the vehicle. Values are mean±SEM. P not significant; n=6 hearts per group (two-way ANOVA)

T3: 3, 5, 3’ triiodo-L-thyronine; LVDP: left ventricular developed pressure; HR: heart rate; CPP: coronary perfusion pressure; +dP/dt_max_: first derivative of ventricular pressure maximum positive; (−dP/dt_min_) first derivative of ventricular pressure minimum negative

The isolated hearts that received T3 intracoronary perfusion (either 1 nM or 100 nM) did not modify LVDP, +dP/dt_máx_, -dP/dt_min_, HR, and CPP when compared with vehicle-perfused isolated hearts (Figure 6). T3 intracoronary perfusion in isolated rat hearts, in concentrations of 1 nM and 100 nM, has been reported to exert an acute positive inotropic effect and a decrease in CPP several seconds or minutes after administration (24-26). Here, contrariwise, we present evidence for the absence of a direct and rapid stimulatory effect of T3 in the isolated normal rat heart.

***Time-dependent phosphorylation patterns of Akt and ERK1/2 elicited by 3, 5, 3’ triiodo-L-thyronine***

**Figure 7:**
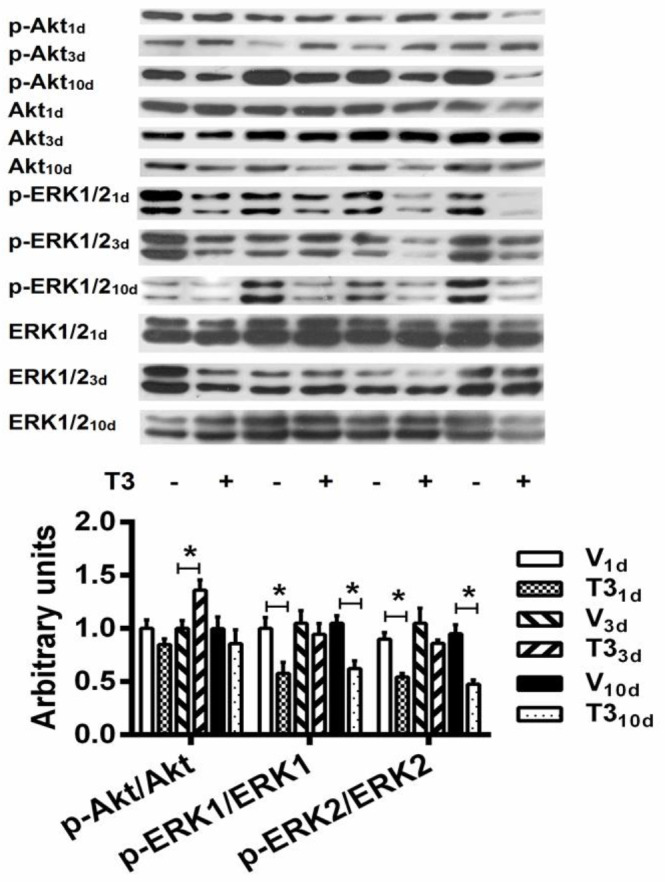
Short-term *in vivo* 3, 5, 3’ triiodo-L-thyronine treatment increases Akt and decreases ERK1/2 signaling. (top) Representative Western blots. (bottom) Densitometric assessment in arbitrary units of the ratios p-Akt/Akt, p-ERK1/ERK1, and p-ERK2/ERK2 in the left ventricles of short-term T3 treated and vehicle-treated rats. Short-term hyperthyroidism was induced by daily subcutaneous injection of T3 (500 μg.kg^-1^) for 1 (T3_1d_), 3 (T3_3d_), and 10 (T3_10d_) days. Control animals were injected daily with the vehicle for 1 (V_1d_), 3 (V_3d_), and 10 (V_10d_) days. Values are the means±SEM. *P<0.05 vs V respective in time; n=4 hearts per group (one-way ANOVA with Tukey’s *post hoc* test)

T3: 3, 5, 3’ triiodo-L-thyronine; V: vehicle; p-Akt/Akt: phosphorylated Akt to total Akt ratio; p-ERK1/ERK1: phosphorylated ERK1 to total ERK1 ratio; p-ERK2/ERK2: phosphorylated ERK2 to total ERK2 ratio

In the left ventricles of T3_1d_ and T3_10d_ rats, p-ERK2 and p-ERK1 decreased significantly, but in those of T3_3d_ rats, p-ERK1/2 did not change in a significant manner, compared with their respective temporal controls (Figure 7). Compared correspondingly with left ventricular tissues from V_1d_, V_3d_, and V_10d_ rats, p-Akt levels were significantly augmented in the left ventricles of T3_3d_ rats and remained unaltered in those of T3_1d_ and T3_10d_ rats (Figure 7). There were no significant differences in total Akt and total ERK1/2 in the cardiac tissues of T3_1d_, T3_3d_, and T3_10d_ rats associated with time-matched controls (Figure 7, densitometric analysis not shown). 

**Figure 8:**
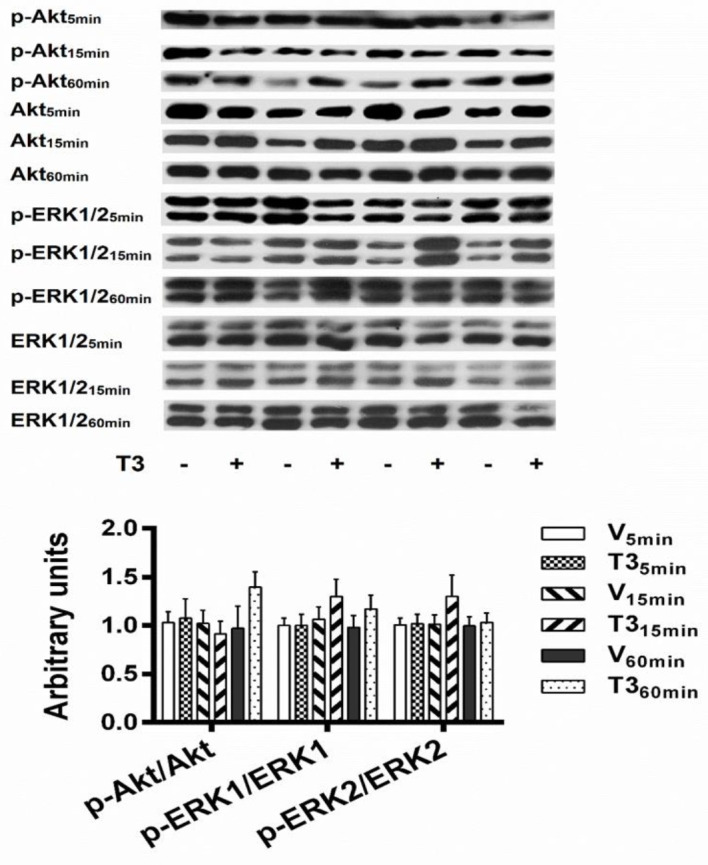
Lack of effects of 3, 5, 3’ triiodo-L-thyronine intracoronary perfusion on Akt and ERK1/2 signaling. (top) Representative Western blots. (bottom) Densitometric assessment in arbitrary units of the ratios of p-Akt/Akt, p-ERK1/ERK1, and p-ERK2/ERK2 in the left ventricles of isolated hearts previously perfused with T3 (100 nM) or vehicle during either 5, 15, or 60 min. Values are the means±SEM. *P* not significant; n=4 hearts per group (one-way ANOVA)

T3: 3, 5, 3’ triiodo-L-thyronine; V: vehicle; p-Akt/Akt: phosphorylated Akt to total Akt ratio; p-ERK1/ERK1: phosphorylated ERK1 to total ERK1 ratio; p-ERK2/ERK2: phosphorylated ERK2 to total ERK2 ratio

The left ventricular levels of Akt, p-Akt, ERK1/2, and p-ERK1/2 at 5, 15, and 60 min of T3 (100 nM) or vehicle intracoronary perfusion to isolated hearts are shown in Figure 8. The expression levels of p-ERK1 and p-ERK2 in response to T3 or vehicle intracoronary perfusion were similar at all established times (Figure 8). 

Also, p-Akt levels at 5, 15, and 60 min of T3 perfusion were analogous to vehicle perfused temporal controls, correspondingly (Figure 8). There were no significant changes in the left ventricular expression levels of total ERK1/2 and total Akt at 5, 15, and 60 min of T3 intracoronary perfusion in isolated hearts, when respectively compared with vehicle-perfused hearts (Figure 8, densitometric analysis not shown). 

## Discussion

Taken together, the data indicate that the thyroid hormone T3, perfused to isolated hearts for as long as one hour, did not modify cardiodynamics and CPP, compared in due time with vehicle perfused hearts. Moreover, there were no significant changes in p-ERK1/2 and p-Akt levels in the left ventricular tissues after 5, 15, and 60 min of T3 intracoronary perfusion. Thus, this lack of functional and molecular effects does not allow us to hypothesize the activation of rapid non-genomic mechanisms by T3 in isolated rat hearts. The evidence shows, chiefly, that T3 has not a direct rapid influence on the activation of pro-survival pathways, Akt and ERK1/2, in the normal heart. 

One main finding of this study was that in the period between 1 and 10 days of treatment with high doses of T3 to induce hyperthyroidism in rats, the hearts that showed hypertrophy and functional improvement (i.e., positive inotropic and lusitropic effects) displayed ERK1/2 inhibited cyclically and Akt activated transiently. Taking into account that the inhibitory changes (dephosphorylations) on ERK1/2 activity on the first and tenth days coincided with normal values of p-Akt and that the increased p-Akt on the third day was paired with normal p-ERK1/2 levels, it is not plausible to infer the existence of a functional interaction between these molecules in response to the thyroid hormone. 

We were interested in elucidating whether T3 intracoronary perfusion in the isolated rat heart has a direct rapid influence on the activation of Akt and ERK1/2 pathways in normal left ventricular tissues. In this experimental condition, however, no changes were detected in the activation levels of Akt and ERK1/2. We did not find articles on the temporal evolution of Akt and ERK1/2 phosphorylations in isolated hearts of normal animals, perfused with THs. However, a Langendorff rat heart model study of ischemia-reperfusion reported that T3 (60 nM) induces cardioprotection by dephosphorylation of the pro-apoptotic signaling mediator, p38 MAPK, at 5, 15, and 60 min of reperfusion compared with vehicle-reperfused hearts (14). Under the same experimental conditions, T3 administration at reperfusion did not modify p-ERK1/2 levels while p-Akt values were significantly lower at 5 min but no significant differences were observed at 15 and 60 min (14). The meaning of this brief p-Akt reduction was not clarified; however, increased - and not diminished - p-Akt levels have been related to pro-survival benefits of THs (9, 10). When studies carried out with neonatal and adult cardiomyocytes are considered, responses of rapid development and brief or sustained duration, on the activation of ERK1/2 and Akt by T3, can be discriminated. In adult rat ventricular cardiomyocyte cultures, T3 (100 nM) stimulated the phosphorylations of ERK1/2 and Akt that were transient with maxima levels in approximately 5 min and disappearance in 10 and 30 min, respectively (27). In neonatal rat ventricular cardiomyocytes (NRVCM), T3 (100 nM) induced an increase in p-ERK levels at 8 min which, within 60 min, returned to the levels of the untreated cells; while no changes in p-Akt levels were found (28). Also, in NRVCM, increased phosphorylation of Akt was detected at 15 min after the addition of T3 (10 nM) and was maintained up to 24 hr (29). These studies with cardiomyocytes are not consistent between them; however, they support the possibility of observing rapid and transient, or sustained, increases in Akt and/or ERK1/2 phosphorylations induced by T3 in isolated hearts. Yet, emphasizing, our results established that T3 intracoronary perfusion has not a direct rapid influence on the activation levels of Akt and ERK of normal rat hearts. 

The time course of transient p-Akt augmentation in the hearts of short-term T3 treated rats (p-Akt levels augmented in left ventricles of T3_3d_ and stayed unaltered in those of T3_1d_ and T3_10d_ rats) approximately agrees in latency and duration with the results obtained with cardiac ventricles of T4 treated mice (12) and NRVCM treated with T3 (12) when they are considered integrally. The expression and phosphorylation of Akt and ERK signaling pathways were examined in left ventricular cardiac tissues of T4 treated mice (12). Phosphorylation of Akt was significantly increased at 3 days and returned to normal levels between days 8 and 14 of T4 treatment. On day 30, there were no changes in the phosphorylation of Akt but there was a significant increase in phosphorylated ERK with T4 treatment. Earlier activation of ERK was not detected. When NRVCM were treated with T3 (100 nM) for 4 days, there was an increase in the phosphorylation of Akt but ERK signaling was not affected (12). These data, thus, support our finding of the delayed and transient increase of p-Akt with a similar time course but contradict our discovery of the cyclic ERK1/2 inhibition (p-ERK1/2 decreased in LV tissues of T3_1d_ and T3_10d_ but not in those of T3_3d_ rats). In this work, the coincidence of an increase in p-Akt in the left ventricles with a time course similar to that reported in the left ventricles of hyperthyroid mice, in which the transient increase in Akt was also linked to cardiac hypertrophy and enhanced function (12), suggests by analogy a causal relationship between transient Akt activation and hypertrophy in the rat heart. 

Otherwise, the inhibition of ERK1/2 activity has been reported in cardiac tissues of short-term T4 and T3 treated mice (13). In T4 treated mice for 2 wk, ventricular cardiac tissues presented decreased p-ERK levels with respect to control tissues (13). Additionally, 2 wk T3 treatment reduced the already increased p-ERK1/2 levels in pressure-overloaded hypertrophied mice hearts, by stimulation of the PI3K/Akt pathway (13). However, the activation state of ERK1/2 and Akt was not defined at different time intervals; it is important to consider, also, that ERK inhibition was measured in mouse ventricular tissue whereas Akt activation was not investigated in the heart. The existence of an interaction between Raf/ERK and PI3K/AKT pathways in response to T3 was probed in neonatal rat cardiac myocytes, and Akt activation was inferred from data obtained with a specific PI3K inhibitor applied to these cells (13). Hence, our results provide important information about activation of Akt and inhibition of ERK1/2 in the left ventricle of the rat heart, depending on the time of treatment with T3.

Arguably, cardiac hypertrophy can be separated into physiological and pathological types (30-32). There is substantial evidence that ERK1/2 and PI3K/Akt are required for promoting the cardiac growth response (31, 33). However, the ERK1/2 pathway is supposed to be a fundamental contributor to pathological hypertrophy, and the PI3K/Akt pathway is considered involved in physiological hypertrophy development (34, 35). The evidence as well supports that physiological cardiac hypertrophy can be induced by increased THs actions (3, 8, 24); nevertheless, it has been proposed that THs-induced cardiac hypertrophy is linked to an initial increase, followed by a pathological reduction, in cardiac function (11, 36). Consequently, it is conceivable that THs may activate signaling pathways that initially produce a phenotype compatible with physiological hypertrophy and, over time, a phenotype well-matched with pathological hypertrophy. However, we must bear in mind that Akt and ERK1/2 cascades have been activated by THs in cardiomyocyte cultures and normal or injured hearts with inconsistent temporal courses that cannot, as a whole, explain the progression of cardiac hypertrophy (12, 14, 27-29, 37-40). To contribute to resolving this matter, we focused on the effects of T3 on the temporal course of ERK1/2 and Akt phosphorylations during the development of cardiac hypertrophy. We found that short-term *in vivo* T3 treatment – but not intracoronary perfusion of T3 to isolated hearts – is associated with transient Akt activation that could indicate a physiological hypertrophy signaling and cyclic ERK1/2 inhibition that could imply deactivation of a pathological hypertrophy signaling, in rat hearts. Nevertheless, the temporal courses of transient activation of Akt and cyclic inhibition of ERK1/2 did not allow surmising an interaction between these pathways. Undoubtedly, this important signaling issue is still far from being resolved.

## Conclusion

Cardiodynamic, hemodynamic, and molecular effects were not observed in T3 perfused hearts; particularly, T3 intracoronary perfusion did not influence Akt and ERK1/2 signaling in normal left ventricular tissues. Hence, the rapid non-genomic effects of T3 were not evident. Our results showed that short-term *in vivo* T3 treatment can develop cardiac hypertrophy concurrent with increased left ventricular function and associated with transient Akt activation and cyclic ERK1/2 inhibition; which implies, in close temporal proximity, activation of a physiological hypertrophy signaling and deactivation of a pathological hypertrophy signaling, respectively. However, based on the temporal courses of transient activation of Akt and cyclic inhibition of ERK1/2 an interaction between both pathways, was not validated. Finally, we propose that the mechanisms responsible for T3 promoted cardiac effects are possibly dependent on its orchestrated actions on the studied molecular pathways, which require further exploration.

This work helps to substantiate the importance of studying the temporal courses of activation or inhibition of Akt and ERK1/2 signaling pathways in the heart. Our general proposition is that the temporal course of activation of signaling molecules must be defined to convincingly establish their causal relationship with certain physiological and pathophysiological events. This knowledge is fundamental to design potential therapeutic strategies aiming to preserve tissular integrity. 
